# Leadership among Directors of Social Work in Long Term Care Facilities

**DOI:** 10.1093/geroni/igab046.3269

**Published:** 2021-12-17

**Authors:** John Paul Abenojar

**Affiliations:** Walden University, Arlington, Virginia, United States

## Abstract

Long term care facilities (LTC) provide ongoing care for seniors and chronically ill. To maximize the quality of the care, LTC staff must be properly trained to respond to patient care crises and communicate across departments. Although researchers have studied the leadership styles, strategies and interactions of facility administrators and nursing directors there is a substantial gap in the literature on the leadership styles and strategies employed by Directors of Social Work (DSW). The aim of this phenomenological study was to address this gap in research by exploring how DSW influenced leadership policies, prepared subordinates for crisis intervention and management, perceived that social workers influence decision making in patient care, and believed that communication amongst LTC staff about patient care could be improved.

